# Supplementary material for: Integrative polygenic risk score improves the prediction accuracy of complex traits and diseases
Source: medRxiv. 2023 Feb 26:2023.02.21.23286110. Preprint. [Version 1] doi: 10.1101/2023.02.21.23286110 (PMC9980241; doi:10.1101/2023.02.21.23286110)
Supplement: Supplementary file 1 [file media-2.docx]

Supplementary Information

**Integrative polygenic risk score improves prediction accuracy of** 

**complex traits and diseases**

Buu Truong et al.

**Table of Contents:**

Supplementary Figure 1. Prediction accuracy of the best PGS catalog versus PRSmix and PRSmix+ in the European ancestry.	3

Supplementary Figure 2. Prediction accuracy of the best PGS catalog versus PRSmix and PRSmix+ in the South Asian ancestry.	4

Supplementary Figure 3. Prediction accuracy of the PRSmix versus PRSmix+ in the European and South Asian ancestries.	5

Supplementary Figure 4. Predictive improvement of PRSmix and PRSmix+ in European and South Asian ancestries.	6

Supplementary Figure 5. Prediction accuracy in the training set of the contributing PGSs to PRSmix+ of coronary artery disease in the European ancestry.	7

Supplementary Figure 6. Empirical prediction accuracy with various training sample sizes for linear combination for coronary artery disease in the European and South Asian ancestries.	8

Supplementary Figure 7. Prediction accuracy in the training set of the contributing PGSs to PRSmix+ of coronary artery disease in the South Asian ancestry.	9

Supplementary Figure 8. Contributing traits to PRSmix+ of stroke in the European ancestry.	10

Supplementary Table 1. Demographics of European and South Asian ancestries in All of Us and Genes and Health cohorts, respectively.	11

Supplementary Table 2. Prediction accuracy of the best PGS catalog, PRSmix and PRSmix+ for 50 traits in the European ancestry from the All of Us cohort.	12

Supplementary Table 3. Prediction accuracy of the best PGS catalog, PRSmix and PRSmix+ for 31 traits in the South Asian ancestry from the Genes and Health cohort.	13

Supplementary Table 4. Comparisons of PRSmix and PRSmix+ to the best PGS Catalog across groups of traits.	15

Supplementary Table 5. Net reclassification improvement of the best PGS Catalog, PRSmix and PRSmix+ compared to the baseline model with clinical risk factors.	16

Supplementary Table 6. Incremental AUC for CAD PRSs in European and South Asian ancestries.	17

Supplementary Table 7. Risk stratification for CAD with PRSs in European and South Asian ancestries.	18

Supplementary Table 8. PheWAS analysis of the best PRS estimated from PGS Catalog and PRSmix+ with disease phenotypes for European ancestry in the All of Us cohort.	19

Supplementary Table 9. Mixing weights of PGS Catalog scores with PRSmix in European ancestry	20

Supplementary Table 10. Mixing weights of PGS Catalog scores with PRSmix+ in European ancestry	20

Supplementary Table 11. Mixing weights of PGS Catalog scores with PRSmix in South Asian ancestry	20

Supplementary Table 12. Mixing weights of PGS Catalog scores with PRSmix+ in South Asian ancestry	20

Supplementary Table 13. Phenotype Definitions and Descriptions from the All of Us Research Program.	21

Supplementary Table 14. Phenotype Definitions and Descriptions from the Genes and Health cohort.	25

###### **Supplementary Figure 1. Prediction accuracy of the best PGS catalog versus PRSmix and PRSmix+ in the European ancestry.** 

The prediction accuracies of the best PGS Catalog are displayed on the x-axis versus PRSmix **(a)** and PRSmix+ **(b)** on the y-axis. The prediction accuracies are estimated as partial R2 and liability R2 for continuous traits and binary traits, respectively. The points represent the mean prediction and the whiskers demonstrate 95% confidence intervals. The black line represents the reference that the prediction accuracy of PGS Catalog is equal to PRSmix and PRSmix+.

###### **Supplementary Figure 2. Prediction accuracy of the best PGS catalog versus PRSmix and PRSmix+ in the South Asian ancestry.** 

The prediction accuracies of the best PGS Catalog are displayed on the x-axis versus PRSmix **(a)** and PRSmix+ **(b)** on the y-axis. The prediction accuracies are estimated as partial R2 and liability R2 for continuous traits and binary traits, respectively. The points represent the mean prediction and the whiskers demonstrate 95% confidence intervals. The black line represents the reference that the prediction accuracy of PGS Catalog is equal to PRSmix and PRSmix+.

###### **Supplementary Figure 3. Prediction accuracy of the PRSmix versus PRSmix+ in the European and South Asian ancestries.** 

The prediction accuracies of the PRSmix are displayed on the x-axis versus PRSmix+ on the y-axis in **a)** European ancestry and **b)** South Asian ancestry. The prediction accuracies are estimated as partial R2 and liability R2 for continuous traits and binary traits, respectively. The points represent the mean prediction and the whiskers demonstrate 95% confidence intervals. The black line represents the reference that the prediction accuracy of PGS Catalog is equal to PRSmix and PRSmix+.

###### **Supplementary Figure 4. Predictive improvement of PRSmix and PRSmix+ in European and South Asian ancestries.** 

The prediction accuracies of the best PGS Catalog are displayed on the x-axis versus ratio of R^2^_PRSmix_ and R^2^_PRSmix+_ on the y-axis. The prediction accuracies are estimated as partial R2 and liability R2 for continuous traits and binary traits, respectively. The red and blue line represents the local regression line for improvement of PRSmix and PRSmix+ over the best PGS Catalog, respectively.

###### **Supplementary Figure 5. Prediction accuracy in the training set of the contributing PGSs to PRSmix+ of coronary artery disease in the European ancestry.** 

We used Elastic Net to estimate the mixing weights of multiple PGS and report the contributing PGS with non-zero effects. Prediction accuracy was estimated with liability R2. The boxes represent the mean prediction accuracy across the traits in that group and the whiskers demonstrate 95% confidence intervals.

###### **Supplementary Figure 6. Empirical prediction accuracy with various training sample sizes for linear combination for coronary artery disease in the European and South Asian ancestries.** 

We assessed the performance of PRSmix and PRSmix+ with various training sample sizes. The prediction accuracy was estimated as liability R2. The points represent the mean prediction accuracy and the whiskers demonstrate 95% confidence intervals.

###### **Supplementary Figure 7. Prediction accuracy in the training set of the contributing PGSs to PRSmix+ of coronary artery disease in the South Asian ancestry.** 

We used Elastic Net to estimate the mixing weights of multiple PGS and report the contributing PGS with non-zero effects. Prediction accuracy was estimated with liability R2. The boxes represent the mean prediction accuracy across the traits in that group and the whiskers demonstrate 95% confidence intervals.

###### **Supplementary Figure 8. Contributing traits to PRSmix+ of stroke in the European ancestry.** 

We used Elastic Net to estimate the mixing weights of multiple PGS and report the contributing PGS with non-zero effects. Prediction accuracy was estimated with liability R2. The boxes represent the mean prediction accuracy across the traits in that group and the whiskers demonstrate 95% confidence intervals.

###### **Supplementary Table 1. Demographics of European and South Asian ancestries in All of Us and Genes and Health cohorts, respectively.** 

ALP, Alkaline Phosphatase; ALT, Alanine transaminase; AST, Aspartate aminotransferase; BMI, Body mass index; Breast CA, Breast cancer; BUN, Blood urea nitrogen; CAD, Coronary artery disease; DBP, Diastolic blood pressure; Hb, Hemoglobin; Hct, Hematocrits; HDL-C, High-density lipoprotein; HTN, Hypertension; LDL-C, Low-density lipoprotein; Prostate CA, Prostate cancer; RA, Rheumatoid arthritis; RBC, Red blood counts; RDW, Red Cell Distribution Width; SBP, Systolic blood pressure; Skin CA, Skin cancer; T2D, Type 2 diabetes; VTE, venous thromboembolism; WBC, White blood counts. NA, not applicable in Genes & Health cohort. 

|  | **European** | **South Asian** |
| --- | --- | --- |
| N | 47894 | 44396 |
| Age (mean (SD)) | 57.63 (16.83) | 40.96 (14.05) |
| Sex = Male (%) | 19118 (39.9) | 24388 (55.2) |
| BMI, kg/m2 (mean (SD)) | 29.13 (7.11) | 27.14 (4.96) |
| Height, cm (mean (SD)) | 169.12 (9.59) | 161.81 (9.05) |
| SBP, mmHg (mean (SD)) | 125.28 (16.40) | 120.38 (11.83) |
| DBP, mmHg (mean (SD)) | 76.68 (10.50) | 75.29 (7.12) |
| HDL-C, mg/dL (median [IQR]) | 54.00 [43.00, 67.00] | 50.27 [42.54, 59.17] |
| LDL-C, mg/dL (median [IQR]) | 107.00 [84.29, 132.86] | 127.61 [104.41, 153.91] |
| Total cholesterol, mg/dL (median [IQR]) | 187.00 [161.00, 216.00] | 208.82 [181.75, 239.75] |
| Triglycerides, mg/dL (median [IQR]) | 102.00 [72.00, 147.00] | 181.57 [120.46, 267.48] |
| A1c (mean (SD)) | 6.36 (1.44) | 6.11 (1.15) |
| Albumin, g/dL (median [IQR]) | 4.10 [3.80, 4.40] | 4.45 [4.28, 4.62] |
| ALT, IU/L (median [IQR]) | 21.00 [17.00, 26.00] | 22.07 [16.25, 31.23] |
| AST, IU/L (median [IQR]) | 20.00 [15.00, 28.00] | 21.83 [18.20, 27.00] |
| ALP, U/L (median [IQR]) | 71.00 [57.00, 88.00] | 80.67 [66.33, 101.49] |
| Bilirubin, mg/dL (median [IQR]) | 0.50 [0.40, 0.70] | NA |
| BUN, mg/dL (median [IQR]) | 15.00 [12.00, 19.00] | 12.35 [10.40, 14.72] |
| Creatinine, mg/dL (median [IQR]) | 0.86 [0.74, 1.00] | 0.84 [0.71, 1.00] |
| Total protein, g/dL (median [IQR]) | 7.00 [6.70, 7.40] | 7.38 [7.13, 7.61] |
| Hb, g/dL (median [IQR]) | 13.50 [12.50, 14.50] | 7.90 [5.21, 11.39] |
| RBC, x10^6/uL (median [IQR]) | 4.48 [4.13, 4.80] | 4.76 [4.41, 5.17] |
| RDW, % (median [IQR]) | 13.30 [12.70, 14.00] | 13.57 [12.96, 14.40] |
| Hct, % (median [IQR]) | 40.60 [37.70, 43.30] | 39.86 [37.00, 43.50] |
| WBC, x10^3/uL (median [IQR]) | 6.80 [5.50, 8.60] | 7.90 [6.84, 9.13] |
| Neutrophils, x10^3/uL (median [IQR]) | 4.30 [3.20, 6.00] | 4.55 [3.69, 5.66] |
| Basophils, x10^3/uL (median [IQR]) | 0.02 [0.00, 0.10] | 0.02 [0.00, 0.05] |
| Eosinophils, x10^3/uL (median [IQR]) | 0.11 [0.10, 0.20] | 0.21 [0.14, 0.32] |
| Monocytes, x10^3/uL (median [IQR]) | 0.55 [0.40, 0.70] | 0.56 [0.47, 0.66] |
| Platelets, x10^3/uL (median [IQR]) | 236.00 [196.00, 283.00] | 266.67 [229.76, 307.78] |
| CAD  (%) | 3090 ( 8.3) | 2929 (6.6) |
| T2D  (%) | 3782 ( 9.6) | 10631 (24.1) |
| HTN  (%) | 17170 (36.3) | 11822 (26.8) |
| Stroke  (%) | 1459 ( 3.7) | 707 (1.6) |
| Asthma  (%) | 8063 (17.0) | 6575 (14.9) |
| Current smoker  (%) | 5810 (12.5) | 3805 ( 8.6) |
| Atrial fibrillation  (%) | 3482 ( 7.4) | 565 (1.3) |
| Depression  (%) | 13661 (28.9) | 9571 (21.7) |
| Glaucoma  (%) | 3057 ( 6.5) | 714 (1.6) |
| Gout  (%) | 1805 ( 3.8) | 877 (2.0) |
| High cholesterol  (%) | 19201 (40.6) | NA |
| Hypothyroid  (%) | 5528 (11.7) | NA |
| Migraine  (%) | 7615 (16.1) | 5184 (11.7) |
| Osteoporosis  (%) | 4718 (10.0) | 448 (1.0) |
| RA  (%) | 2002 ( 4.2) | 1274 ( 2.9) |
| VTE  (%) | 2021 ( 4.3) | 477 (1.1) |
| Breast CA  (%) | 2187 ( 7.7) | 577 (1.3) |
| Prostate CA  (%) | 1311 ( 6.9) | 117 (0.3) |
| Skin CA  (%) | 6048 (12.8) | 66 (0.1) |
| Melanoma  (%) | 2604 ( 5.5) | 31 (0.1) |

###### **Supplementary Table 2. Prediction accuracy of the best PGS catalog, PRSmix and PRSmix+ for 50 traits in the European ancestry from the All of Us cohort.**

The prediction accuracy was assessed as partial R2 and liability R2 for continuous and binary traits, respectively. The partial R2 is a difference of R2 between the model with PRS and covariates including age, sex, and 10 PCs versus the base model with only covariates. Prediction accuracy for binary traits was assessed with liability R2 where disease prevalence was approximately estimated as a proportion of cases in the testing set. ALP, Alkaline Phosphatase; ALT, Alanine transaminase; AST, Aspartate aminotransferase; BMI, Body mass index; Breast CA, Breast cancer; BUN, Blood urea nitrogen; CAD, Coronary artery disease; DBP, Diastolic blood pressure; Hb, Hemoglobin; Hct, Hematocrits; HDL-C, High-density lipoprotein; HTN, Hypertension; LDL-C, Low-density lipoprotein; Prostate CA, Prostate cancer; RA, Rheumatoid arthritis; RBC, Red blood counts; RDW, Red Cell Distribution Width; SBP, Systolic blood pressure; Skin CA, Skin cancer; T2D, Type 2 diabetes; VTE, venous thromboembolism; WBC, White blood counts. NA, not applicable in Genes & Health cohort. 

| **Trait** | **Subtypes** | **Best PGS Catalog** **(95% CI)** | **PRSmix** **(95% CI)** | **PRSmix+** **(95% CI)** | **N** | **Disease prevalence** |
| --- | --- | --- | --- | --- | --- | --- |
| Height | Anthropometric | 0.169 (0.155-0.183) | 0.195 (0.18-0.21) | 0.196 (0.181-0.21) | 45229 | NA |
| BMI | Anthropometric | 0.125 (0.113-0.138) | 0.147 (0.133-0.161) | 0.156 (0.142-0.17) | 44837 | NA |
| Platelets | Blood counts | 0.084 (0.07-0.098) | 0.119 (0.103-0.136) | 0.126 (0.109-0.142) | 26709 | NA |
| Monocytes | Blood counts | 0.08 (0.063-0.098) | 0.101 (0.082-0.12) | 0.115 (0.095-0.134) | 17935 | NA |
| RBC | Blood counts | 0.065 (0.052-0.078) | 0.072 (0.058-0.085) | 0.075 (0.061-0.089) | 25818 | NA |
| RDW | Blood counts | 0.06 (0.047-0.073) | 0.073 (0.059-0.087) | 0.093 (0.077-0.108) | 25300 | NA |
| WBC | Blood counts | 0.058 (0.046-0.071) | 0.068 (0.055-0.082) | 0.079 (0.065-0.093) | 25590 | NA |
| Eosinophils | Blood counts | 0.057 (0.042-0.072) | 0.074 (0.058-0.091) | 0.082 (0.065-0.1) | 17321 | NA |
| Neutrophils | Blood counts | 0.039 (0.026-0.052) | 0.04 (0.027-0.053) | 0.049 (0.035-0.063) | 17474 | NA |
| Hb | Blood counts | 0.026 (0.017-0.034) | 0.03 (0.021-0.039) | 0.033 (0.023-0.042) | 26626 | NA |
| Hct | Blood counts | 0.021 (0.014-0.029) | 0.026 (0.018-0.035) | 0.027 (0.019-0.036) | 25873 | NA |
| Basophils | Blood counts | 0.014 (0.006-0.022) | 0.014 (0.006-0.022) | 0.028 (0.017-0.039) | 16927 | NA |
| Skin CA | Cancer | 0.037 (0.029-0.044) | 0.047 (0.038-0.055) | 0.063 (0.053-0.072) | 47314 | 0.128 |
| Breast CA | Cancer | 0.043 (0.033-0.053) | 0.054 (0.043-0.065) | 0.054 (0.043-0.065) | 28402 | 0.077 |
| Prostate CA | Cancer | 0.072 (0.056-0.088) | 0.087 (0.07-0.104) | 0.087 (0.07-0.104) | 18912 | 0.069 |
| Melanoma | Cancer | 0.013 (0.009-0.018) | 0.014 (0.009-0.019) | 0.03 (0.023-0.037) | 47314 | 0.055 |
| HDL-C | Cardiometabolic | 0.106 (0.088-0.124) | 0.128 (0.109-0.148) | 0.151 (0.13-0.171) | 20000 | NA |
| Triglycerides | Cardiometabolic | 0.092 (0.075-0.109) | 0.113 (0.094-0.131) | 0.126 (0.107-0.145) | 20497 | NA |
| LDL-C | Cardiometabolic | 0.059 (0.045-0.074) | 0.074 (0.058-0.089) | 0.078 (0.062-0.094) | 20131 | NA |
| Total cholesterol | Cardiometabolic | 0.057 (0.044-0.071) | 0.068 (0.053-0.083) | 0.08 (0.064-0.096) | 20966 | NA |
| DBP | Cardiometabolic | 0.032 (0.025-0.039) | 0.042 (0.034-0.05) | 0.049 (0.04-0.058) | 45314 | NA |
| SBP | Cardiometabolic | 0.024 (0.018-0.03) | 0.029 (0.022-0.036) | 0.034 (0.027-0.042) | 45314 | NA |
| High cholesterol | Cardiometabolic | 0.03 (0.023-0.036) | 0.038 (0.03-0.046) | 0.046 (0.038-0.055) | 47314 | 0.406 |
| HTN | Cardiometabolic | 0.045 (0.037-0.053) | 0.064 (0.054-0.073) | 0.076 (0.066-0.086) | 47314 | 0.363 |
| T2D | Cardiometabolic | 0.089 (0.077-0.101) | 0.11 (0.097-0.123) | 0.125 (0.111-0.139) | 39397 | 0.096 |
| CAD | Cardiometabolic | 0.019 (0.013-0.025) | 0.029 (0.022-0.037) | 0.036 (0.028-0.044) | 37329 | 0.083 |
| Atrial fibrillation | Cardiometabolic | 0.034 (0.027-0.041) | 0.037 (0.03-0.045) | 0.042 (0.034-0.05) | 47314 | 0.074 |
| Stroke | Cardiometabolic | 0.009 (0.005-0.013) | 0.009 (0.005-0.013) | 0.022 (0.015-0.028) | 39445 | 0.06 |
| VTE | Cardiometabolic | 0.013 (0.008-0.017) | 0.022 (0.016-0.028) | 0.035 (0.028-0.043) | 47314 | 0.043 |
| Bilirubin | Biochemistry | 0.162 (0.143-0.18) | 0.165 (0.147-0.184) | 0.17 (0.151-0.189) | 25787 | NA |
| ALP | Biochemistry | 0.107 (0.091-0.123) | 0.126 (0.11-0.143) | 0.139 (0.122-0.156) | 26759 | NA |
| Total protein | Biochemistry | 0.044 (0.033-0.054) | 0.047 (0.036-0.058) | 0.047 (0.036-0.059) | 26655 | NA |
| A1c | Biochemistry | 0.043 (0.024-0.063) | 0.049 (0.028-0.07) | 0.08 (0.054-0.106) | 7718 | NA |
| Creatinine | Biochemistry | 0.04 (0.03-0.05) | 0.042 (0.032-0.053) | 0.052 (0.041-0.063) | 28793 | NA |
| BUN | Biochemistry | 0.018 (0.011-0.024) | 0.019 (0.012-0.026) | 0.022 (0.014-0.029) | 28804 | NA |
| Albumin | Biochemistry | 0.017 (0.01-0.024) | 0.019 (0.012-0.027) | 0.03 (0.021-0.04) | 24399 | NA |
| AST | Biochemistry | 0.011 (0.005-0.017) | 0.011 (0.005-0.017) | 0.025 (0.016-0.034) | 23640 | NA |
| ALT | Biochemistry | 0.008 (0.003-0.013) | 0.009 (0.004-0.014) | 0.036 (0.026-0.046) | 26235 | NA |
| Depression | Other conditions | 0.014 (0.009-0.018) | 0.015 (0.01-0.02) | 0.018 (0.013-0.023) | 47314 | 0.289 |
| Asthma | Other conditions | 0.04 (0.032-0.048) | 0.044 (0.036-0.052) | 0.049 (0.041-0.058) | 47314 | 0.17 |
| Migraine | Other conditions | 0.003 (0.001-0.005) | 0.004 (0.001-0.006) | 0.015 (0.01-0.02) | 47314 | 0.161 |
| Current smoker | Other conditions | 0.071 (0.061-0.081) | 0.077 (0.067-0.088) | 0.152 (0.138-0.165) | 46583 | 0.125 |
| Hypothyroid | Other conditions | 0.026 (0.02-0.032) | 0.035 (0.028-0.043) | 0.039 (0.032-0.047) | 47314 | 0.117 |
| Osteoporosis | Other conditions | 0.012 (0.007-0.016) | 0.012 (0.007-0.016) | 0.021 (0.015-0.026) | 47314 | 0.1 |
| Glaucoma | Other conditions | 0.025 (0.019-0.031) | 0.037 (0.029-0.044) | 0.037 (0.029-0.044) | 47314 | 0.065 |
| RA | Other conditions | 0.004 (0.002-0.007) | 0.009 (0.005-0.012) | 0.009 (0.005-0.013) | 47314 | 0.042 |
| Gout | Other conditions | 0.036 (0.028-0.043) | 0.045 (0.037-0.053) | 0.06 (0.05-0.069) | 47314 | 0.038 |

###### **Supplementary Table 3. Prediction accuracy of the best PGS catalog, PRSmix and PRSmix+ for 31 traits in the South Asian ancestry from the Genes and Health cohort.** 

The prediction accuracies for 32 traits were assessed as partial R2 and liability R2 for continuous and binary traits, respectively. Binary traits with disease prevalence > 2% were selected. The partial R2 is a difference of R2 between the model with PRS and covariates including age, sex, and 10 PCs versus the base model with only covariates. Prediction accuracy for binary traits was assessed with liability-R2 where disease prevalence was approximately estimated as a proportion of cases in the testing set. ALP, Alkaline Phosphatase; ALT, Alanine transaminase; AST, Aspartate aminotransferase; BMI, Body mass index; Breast CA, Breast cancer; BUN, Blood urea nitrogen; CAD, Coronary artery disease; DBP, Diastolic blood pressure; Hb, Hemoglobin; Hct, Hematocrits; HDL-C, High-density lipoprotein; HTN, Hypertension; LDL-C, Low-density lipoprotein; Prostate CA, Prostate cancer; RA, Rheumatoid arthritis; RBC, Red blood counts; RDW, Red Cell Distribution Width; SBP, Systolic blood pressure; Skin CA, Skin cancer; T2D, Type 2 diabetes; VTE, venous thromboembolism; WBC, White blood counts. NA, not applicable in Genes & Health cohort. 

| **Trait** | **Subtypes** | **Best PGS Catalog** **(95% CI)** | **PRSmix** **(95% CI)** | **PRSmix+** **(95% CI)** | **N** | **Disease prevalence** |
| --- | --- | --- | --- | --- | --- | --- |
| BMI | Anthropometric | 0.044 (0.034-0.053) | 0.06 (0.049-0.071) | 0.067 (0.056-0.079) | 33223 | NA |
| Height | Anthropometric | 0.075 (0.063-0.088) | 0.088 (0.075-0.101) | 0.089 (0.076-0.102) | 34175 | NA |
| Basophils | Blood counts | 0.017 (0.011-0.022) | 0.018 (0.012-0.024) | 0.03 (0.022-0.038) | 35480 | NA |
| Eosinophils | Blood counts | 0.077 (0.065-0.089) | 0.091 (0.078-0.104) | 0.098 (0.085-0.112) | 35282 | NA |
| Monocytes | Blood counts | 0.107 (0.093-0.121) | 0.13 (0.115-0.144) | 0.138 (0.123-0.153) | 35491 | NA |
| Neutrophils | Blood counts | 0.046 (0.036-0.055) | 0.05 (0.04-0.06) | 0.055 (0.045-0.066) | 35497 | NA |
| Platelets | Blood counts | 0.11 (0.096-0.124) | 0.13 (0.115-0.144) | 0.135 (0.12-0.15) | 35364 | NA |
| RBC | Blood counts | 0.034 (0.025-0.042) | 0.039 (0.031-0.048) | 0.041 (0.031-0.05) | 35277 | NA |
| Hct | Blood counts | 0.014 (0.008-0.02) | 0.016 (0.009-0.022) | 0.017 (0.011-0.024) | 28832 | NA |
| RDW | Blood counts | 0.036 (0.028-0.045) | 0.04 (0.031-0.049) | 0.047 (0.037-0.056) | 34237 | NA |
| WBC | Blood counts | 0.07 (0.059-0.082) | 0.084 (0.072-0.097) | 0.089 (0.076-0.102) | 35290 | NA |
| DBP | Cardiometabolic | 0.031 (0.023-0.038) | 0.04 (0.031-0.048) | 0.046 (0.036-0.055) | 35929 | NA |
| HDL-C | Cardiometabolic | 0.107 (0.092-0.122) | 0.125 (0.109-0.141) | 0.13 (0.114-0.146) | 30235 | NA |
| LDL-C | Cardiometabolic | 0.088 (0.074-0.102) | 0.121 (0.105-0.137) | 0.121 (0.105-0.137) | 28675 | NA |
| SBP | Cardiometabolic | 0.031 (0.023-0.039) | 0.036 (0.027-0.044) | 0.044 (0.035-0.054) | 35941 | NA |
| Total cholesterol | Cardiometabolic | 0.089 (0.075-0.104) | 0.112 (0.097-0.128) | 0.127 (0.111-0.143) | 28657 | NA |
| Triglycerides | Cardiometabolic | 0.091 (0.077-0.105) | 0.108 (0.093-0.123) | 0.112 (0.096-0.127) | 28881 | NA |
| HTN | Cardiometabolic | 0.018 (0.013-0.024) | 0.022 (0.016-0.028) | 0.027 (0.02-0.033) | 44188 | 0.268 |
| T2D | Cardiometabolic | 0.057 (0.047-0.066) | 0.061 (0.051-0.07) | 0.065 (0.055-0.075) | 44188 | 0.241 |
| CAD | Cardiometabolic | 0.006 (0.003-0.009) | 0.014 (0.009-0.019) | 0.02 (0.014-0.026) | 44188 | 0.066 |
| A1c | Biochemistry | 0.048 (0.038-0.058) | 0.053 (0.042-0.063) | 0.071 (0.059-0.084) | 32074 | NA |
| Albumin | Biochemistry | 0.026 (0.019-0.034) | 0.029 (0.021-0.037) | 0.033 (0.025-0.041) | 34324 | NA |
| ALP | Biochemistry | 0.067 (0.055-0.079) | 0.077 (0.065-0.09) | 0.08 (0.068-0.092) | 33978 | NA |
| ALT | Biochemistry | 0.028 (0.02-0.036) | 0.03 (0.022-0.038) | 0.031 (0.023-0.039) | 33974 | NA |
| AST | Biochemistry | 0.025 (0.013-0.037) | 0.03 (0.017-0.042) | 0.03 (0.017-0.042) | 13551 | NA |
| BUN | Biochemistry | 0.013 (0.007-0.018) | 0.014 (0.008-0.019) | 0.016 (0.009-0.022) | 30438 | NA |
| Creatinine | Biochemistry | 0.026 (0.019-0.034) | 0.029 (0.022-0.037) | 0.035 (0.027-0.044) | 34773 | NA |
| Total protein | Biochemistry | 0.053 (0.043-0.064) | 0.058 (0.047-0.069) | 0.059 (0.048-0.07) | 32434 | NA |
| Asthma | Other conditions | 0.06 (0.051-0.07) | 0.065 (0.055-0.075) | 0.069 (0.059-0.08) | 44188 | 0.149 |
| Migraine | Other conditions | 0.004 (0.001-0.006) | 0.004 (0.001-0.006) | 0.011 (0.007-0.016) | 44188 | 0.117 |
| Current smoker | Other conditions | 0.003 (0.001-0.006) | 0.004 (0.001-0.016) | 0.006 (0.002-0.009) | 44188 | 0.085 |
| RA | Other conditions | 0.008 (0.005-0.012) | 0.008 (0.005-0.012) | 0.009 (0.005-0.013) | 44188 | 0.029 |

###### **Supplementary Table 4. Comparisons of PRSmix and PRSmix+ to the best PGS Catalog across groups of traits.** 

We categorized all of 47 traits from European ancestry and 31 traits from South Asian ancestry into 6 main groups including anthropometric, blood counts, cancer, cardiometabolic, biochemistry and other conditions. Cancer phenotypes in South Asian ancestry are omitted due to prevalence < 2%.

|  |  | **European** |  | **South Asian** |  |
| --- | --- | --- | --- | --- | --- |
| **Group** | **Model** | **Ratio** **(95% CI)** | **P-value** | **Ratio** **(95% CI)** | **P-value** |
| Anthropometric | Best PGS Catalog | 1 (1; 1) | Reference | 1 (1; 1) | Reference |
|  | PRSmix | 1.163 (1.144; 1.182) | < 2.2e-16 | 1.276 (1.071; 1.482) | 8.07E-03 |
|  | PRSmix+ | 1.199 (1.114; 1.283) | 3.4e-6 | 1.362 (0.998; 1.727) | 0.0503 |
| Blood counts | Best PGS Catalog | 1 (1; 1) | Reference | 1 (1; 1) | Reference |
|  | PRSmix | 1.19 (1.112; 1.268) | 1.29E-07 | 1.15 (1.112; 1.188) | 4.91E-15 |
|  | PRSmix+ | 1.428 (1.274; 1.582) | 4.28E-08 | 1.323 (1.178; 1.468) | 1.14E-05 |
| Cancer | Best PGS Catalog | 1 (1; 1) | Reference | NA | NA |
|  | PRSmix | 1.192 (1.083; 1.3) | 0.0005 | NA | NA |
|  | PRSmix+ | 1.605 (1.127; 2.083) | 0.0126 | NA | NA |
| Cardiometabolic | Best PGS Catalog | 1 (1; 1) | Reference | 1 (1; 1) | Reference |
|  | PRSmix | 1.293 (1.192; 1.393) | 1.07E-08 | 1.334 (1.084; 1.584) | 0.008 |
|  | PRSmix+ | 1.658 (1.404; 1.913) | 3.56E-07 | 1.563 (1.133; 1.993) | 0.009 |
| Biochemistry | Best PGS Catalog | 1 (1; 1) | Reference | 1 (1; 1) | Reference |
|  | PRSmix | 1.096 (1.061; 1.132) | 7.74E-08 | 1.11 (1.082; 1.137) | 4.66E-15 |
|  | PRSmix+ | 1.848 (1.1; 2.597) | 2.56E-02 | 1.233 (1.155: 1.312) | 5.22E-09 |
| Other conditions | Best PGS Catalog | 1 (1; 1) | Reference | 1 (1; 1) | Reference |
|  | PRSmix | 1.308 (1.104; 1.512) | 2.95E-03 | 1.033 (0.993; 1.073) | 0.1030 |
|  | PRSmix+ | 2.045 (1.116; 2.973) | 0.0374 | 2.096 (0.787; 3.405) | 0.0990 |

###### **Supplementary Table 5. Net reclassification improvement of the best PGS Catalog, PRSmix and PRSmix+ compared to the baseline model with clinical risk factors.** 

The baseline model for risk prediction includes age, sex, total cholesterol, HDL-C, systolic blood pressure, BMI, type 2 diabetes and current smoking status. The clinical risk factors includes age, sex,  EUR, European; SAS, South Asian. NRI, Net Reclassification improvement.

| **Ancestry** | **Methods** | **NRI (95% CI)** | **NRI+ (95% CI)** | **NRI- (95% CI)** | **P** |
| --- | --- | --- | --- | --- | --- |
| **EUR** | PRSmix+^a^ | 0.46 (0.373; 0.547) | 0.201 (0.143; 0.258) | 0.26 (0.217; 0.302) | < 2e-16 |
|  | PRSmix^a^ | 0.376 (0.283; 0.47) | 0.151 (0.089; 0.213) | 0.225 (0.178; 0.272) | 2.19E-15 |
|  | Best PGS Catalog^a^ | 0.266 (0.168; 0.365) | 0.109 (0.043; 0.174) | 0.158 (0.109; 0.207) | 1.06E-07 |
|  | PRSmix+^b^ | 0.345 (0.219; 0.471) | 0.16 (0.082; 0.238) | 0.185 (0.116; 0.254) | 7.08E-08 |
|  | PRSmix^b^ | 0.298 (0.179; 0.418) | 0.126 (0.051; 0.202) | 0.172 (0.108; 0.236) | 9.11E-07 |
|  | Best PGS Catalog^b^ | 0.19 (0.051; 0.328) | 0.068 (-0.019; 0.155) | 0.122 (0.05; 0.194) | 0.006984 |
| **SAS** | PRSmix+^a^ | 0.29 (0.18; 0.404) | 0.098 (0.03; 0.166) | 0.194 (0.132; 0.257) | 2.69E-07 |
|  | PRSmix^a^ | 0.256 (0.158; 0.354) | 0.094 (0.029; 0.16) | 0.161 (0.111; 0.212) | 2.72E-07 |
|  | Best PGS Catalog^a^ | 0.16 (0.061; 0.259) | 0.073 (0.01; 0.137) | 0.087 (0.034; 0.139) | 1.52E-03 |
|  | PRSmix+^b^ | 0.269 (0.163; 0.375) | 0.101 (0.032; 0.17) | 0.168 (0.109; 0.227) | 6.07E-07 |
|  | PRSmix^b^ | 0.257 (0.144; 0.369) | 0.11 (0.034; 0.186) | 0.147 (0.089; 0.204) | 7.18E-06 |
|  | Best PGS Catalog^b^ | 0.13 (0.018; 0.241) | 0.057 (-0.013; 0.127) | 0.073 (0.013; 0.132) | 2.17E-02 |

^a^ model adjusted for age, sex, genotyping array, and the first ten principal components of ancestry.

^b^ model adjusted for age, sex, first ten principal components, total cholesterol, high-density lipoprotein, systolic blood pressure, type 2 diabetes and current smoking status.

###### **Supplementary Table 6. Incremental AUC for CAD PRSs in European and South Asian ancestries.** 

The incremental AUC was estimated as the difference between AUC of the full model of PRS and covariates including age, sex and 10 PCs, and the null model with only covariates. The risk was adjusted for age, sex and 10 PCs. EUR, European; SAS, South Asian.

| **Ancestry** | **Methods** | **Incremental AUC** **(95% CI)** | **P-value** |
| --- | --- | --- | --- |
| **EUR** | Best PGS Catalog | 0.007 (0.007; 0.008) | < 2.22e-16 |
|  | PRSmix | 0.013 (0.013; 0.014) | < 2.22e-16 |
|  | PRSmix+ | 0.019 (0.018; 0.02) | < 2.22e-16 |
| **SAS** | Best PGS Catalog | 0.003 (0.002; 0.003) | < 2.22e-16 |
|  | PRSmix | 0.006 (0.005; 0.007) | < 2.22e-16 |
|  | PRSmix+ | 0.008 (0.007; 0.009) | < 2.22e-16 |

###### **Supplementary Table 7. Risk stratification for CAD with PRSs in European and South Asian ancestries.** 

Odds ratios were calculated by comparing those with high PRS with the remaining of population in a logistic regression in each ancestry. The risk was adjusted for age, sex and 10 PCs. EUR, European; SAS, South Asian.

| **Ancestry** | **Methods** | **OR (95% CI)** | **P-value** |
| --- | --- | --- | --- |
| EUR | Best PGS Catalog^a^ | 2.14 (1.661; 2.737) | 2.267e-09 |
|  | PRSmix^a^ | 2.252 (1.752; 2.876) | 1.319e-10 |
|  | PRSmix+^a^ | 2.537 (1.966; 3.252) | 3.914e-13 |
|  | Best PGS Catalog^b^ | 1.953 (1.392; 2.714) | 8.381e-05 |
|  | PRSmix^b^ | 2.172 (1.553; 3.013) | 4.396e-06 |
|  | PRSmix+^b^ | 2.351 (1.666; 3.293) | 8.587e-07 |
| SAS | Best PGS Catalog^a^ | 1.728 (1.299; 2.277) | 1.310e-04 |
|  | PRSmix^a^ | 2.024 (1.533; 2.648) | 4.155e-07 |
|  | PRSmix+^a^ | 2.343 (1.787; 3.05) | 4.220e-10 |
|  | Best PGS Catalog^b^ | 1.463 (1.053; 2.006) | 2.055e-02 |
|  | PRSmix^b^ | 1.909 (1.402; 2.575) | 2.970e-05 |
|  | PRSmix+^b^ | 2.14 (1.579; 2.876) | 6.389e-7 |

^a^ model adjusted for age, sex, genotyping array, and the first ten principal components of ancestry.

^b^ model adjusted for age, sex, first ten principal components, total cholesterol, high-density lipoprotein, systolic blood pressure, type 2 diabetes and current smoking status.

###### **Supplementary Table 8. PheWAS analysis of the best PRS estimated from PGS Catalog and PRSmix+ with disease phenotypes for European ancestry in the All of Us cohort.** 

The associations of PRSs and disease phecodes were adjusted for age, sex and 10 PCs. Here we display the associations with P < 2.75 x 10^-5^ (​​0.05/1815) among 1815 phecodes.

| **Disease phenotype** | **Best PGS Catalog** |  | **PRSmix+** |  |
| --- | --- | --- | --- | --- |
|  | **OR (95% CI)** | **P-value** | **OR (95% CI)** | **P-value** |
| Ischemic Heart Disease | 1.402 (1.285; 1.531) | 2.90e-14* | 1.607 (1.469; 1.761) | < 2e-16* |
| Coronary atherosclerosis | 1.403 (1.286; 1.532) | 3.05e-14* | 1.603 (1.464; 1.757) | < 2e-16* |
| Essential hypertension | 1.197 (1.136; 1.261) | 1.48e-11* | 1.334 (1.265; 1.407) | < 2e-16* |
| Hypertension | 1.203 (1.142; 1.268) | 3.16e-12* | 1.343 (1.273; 1.416) | < 2e-16* |
| Angina pectoris | 1.633 (1.426; 1.87) | 1.48e-12* | 1.816 (1.576; 2.095) | < 2e-16* |
| Diabetes mellitus | 1.197 (1.117; 1.282) | 3.52e-07* | 1.337 (1.247; 1.435) | 3.33e-16* |
| Type 2 diabetes | 1.203 (1.12; 1.292) | 3.85e-07* | 1.339 (1.245; 1.44) | 2.44e-15* |
| Morbid obesity | 1.159 (1.079; 1.246) | 5.41E-05 | 1.324 (1.231; 1.425) | 4.11e-14* |
| Myocardial infarction | 1.458 (1.272; 1.671) | 7.60e-08* | 1.677 (1.455; 1.936) | 7.85e-13* |
| Disorders of lipoid metabolism | 1.163 (1.104; 1.225) | 1.03e-08* | 1.207 (1.146; 1.272) | 1.06e-12* |
| Hyperlipidemia | 1.165 (1.106; 1.227) | 7.88e-09* | 1.206 (1.145; 1.271) | 1.56e-12* |
| Obesity | 1.131 (1.071; 1.194) | 9.19e-06* | 1.218 (1.153; 1.287) | 1.79e-12* |
| Overweight, obesity and other hyperalimentation | 1.102 (1.048; 1.159) | 0.000148 | 1.176 (1.118; 1.238) | 3.43e-10* |
| Sleep apnea | 1.129 (1.059; 1.203) | 0.000216 | 1.2 (1.124; 1.28) | 3.33e-08* |
| Unstable angina (intermediate coronary syndrome) | 1.464 (1.206; 1.773) | 0.000123 | 1.737 (1.422; 2.124) | 5.91e-08* |
| Other chronic ischemic heart disease, unspecified | 1.383 (1.167; 1.637) | 0.000192 | 1.618 (1.357; 1.932) | 7.47e-08* |
| Hypertensive heart and/or renal disease | 1.171 (1.057; 1.297) | 0.002511 | 1.329 (1.198; 1.476) | 7.76e-08* |
| Obstructive sleep apnea | 1.135 (1.061; 1.215) | 0.000261 | 1.198 (1.118; 1.283) | 2.49e-07* |
| Encounter for long-term (current) use of anticoagulants, antithrombotics, aspirin | 1.111 (1.029; 1.2) | 0.006891 | 1.215 (1.124; 1.313) | 8.08e-07* |
| Mixed hyperlipidemia | 1.096 (1.017; 1.181) | 0.01628 | 1.207 (1.119; 1.302) | 9.66e-07* |
| Tobacco use disorder | 1.142 (1.078; 1.21) | 6.29e-06* | 1.153 (1.087; 1.222) | 1.76e-06* |
| Chronic airway obstruction | 1.207 (1.104; 1.32) | 3.85E-05 | 1.248 (1.139; 1.367) | 1.88e-06* |
| Encounter for long-term (current) use of aspirin | 1.111 (1.028; 1.201) | 0.007912 | 1.209 (1.118; 1.308) | 2.09e-06* |
| Hypercholesterolemia | 1.094 (1.025; 1.167) | 0.006543 | 1.166 (1.092; 1.245) | 3.87e-06* |
| Other symptoms of respiratory system | 1.095 (1.044; 1.149) | 0.000213 | 1.119 (1.067; 1.175) | 4.42e-06* |
| Polyneuropathy in diabetes | 1.229 (1.052; 1.434) | 0.009646 | 1.438 (1.228; 1.683) | 6.26e-06* |
| Abnormal function study of cardiovascular system | 1.298 (1.123; 1.499) | 0.000435 | 1.391 (1.2; 1.612) | 1.14e-05* |
| Atherosclerosis | 1.359 (1.189; 1.552) | 7.01e-06* | 1.349 (1.176; 1.548) | 1.81e-05* |
| Hypertensive chronic kidney disease | 1.195 (1.046; 1.363) | 0.008586 | 1.334 (1.166; 1.527) | 2.73e-05* |
| Other dyspnea | 1.144 (1.075; 1.217) | 2.24e-05* | 1.136 (1.067; 1.209) | 6.41E-05 |

###### **Supplementary Table 9. Mixing weights of PGS Catalog scores with PRSmix in European ancestry**

###### See the Excel file

###### **Supplementary Table 10. Mixing weights of PGS Catalog scores with PRSmix+ in European ancestry**

###### See the Excel file

###### **Supplementary Table 11. Mixing weights of PGS Catalog scores with PRSmix in South Asian ancestry**

###### See the Excel file

###### **Supplementary Table 12. Mixing weights of PGS Catalog scores with PRSmix+ in South Asian ancestry**

###### See the Excel file

###### **Supplementary Table 13. Phenotype Definitions and Descriptions from the All of Us Research Program.** 

For binary phenotypes, cases were curated as per the definitions below.  Controls were defined as those who did not meet the case definition, and who had consented to linking of EHR data. 1.9% of participants (6,692/313,514 (1.9%)) of participants did not consent to EHR data linkage in the V4 Data Release.

Continuous measures included either body measurements, which were directly measured at the participant’s study visit, or laboratory measurements, obtained from electronic health record data.  For all laboratory measurements, if a participant had multiple measurements available, the most recent measurements was used (closest to CDR date) after cleaning.

| **Trait** | **Trait Subtype** | **Variable Type** | **Definition** |
| --- | --- | --- | --- |
| BMI | Anthropometric | Continuous | Calculated BMI from participant measurements obtained during the study visit.  Patients who indicated pregnancy at enrollment were excluded. Unit of measure is kg/m^2.. |
| Height | Anthropometric | Continuous | From participant measurement data obtained during the study visit. Unit of measure is cm. |
| Albumin (plasma/serum) | Biochemistry | Continuous | Obtained from electronic health record lab measurements (LOINC Code: 1751-7; OMOP Concept ID: 3024561). Unit of measurement is g/dL. |
| ALP (plasma/serum) | Biochemistry | Continuous | Obtained from electronic health record lab measurements (LOINC Code: 6768-6; OMOP Concept ID: 3035995). Unit of measurement is units per liter. |
| ALT/SGPT (plasma/serum) | Biochemistry | Continuous | Obtained from electronic health record lab measurements (LOINC Code: 1742-6; OMOP Concept ID: 3006923). Unit of measurement is units per liter. |
| AST/SGOT (plasma/serum) | Biochemistry | Continuous | Obtained from electronic health record lab measurements (LOINC Code: 1920-8; OMOP Concept ID: 3013721). Unit of measurement is units per liter. |
| Blood Urea Nitrogen (BUN) (plasma/serum) | Biochemistry | Continuous | Obtained from electronic health record lab measurements (LOINC Code: 3094-0; OMOP Concept ID: 3013682). Unit of measurement is mg/dL. |
| Creatinine (plasma/serum) | Biochemistry | Continuous | Obtained from electronic health record lab measurements (LOINC Code: 2160-0; OMOP Concept ID: 3016723). Unit of measurement is mg/dL. |
| A1c (blood) | Biochemistry | Continuous | Obtained from electronic health record lab measurements (LOINC Code: LP16413 ; OMOP Code 40789263).  No adjustment for concurrent diabetes medication use.  Unit of measure is %. |
| Total bilirubin (plasma/serum) | Biochemistry | Continuous | Obtained from electronic health record lab measurements (LOINC Code: 1975-2; OMOP Concept ID: 3024128). Unit of measurement is mg/dL. |
| Total Protein  (plasma/serum) | Biochemistry | Continuous | Obtained from electronic health record lab measurements (LOINC Code: 2885-2; OMOP Concept ID: 3020630). Unit of measurement is g/dL. |
| WBC (blood) | Blood counts | Continuous | Obtained from electronic health record lab measurements (LOINC Code: 6690-2; OMOP Concept ID: 3000905). Unit of measurement is thousand/microliter. |
| Basophils (blood) | Blood counts | Continuous | Obtained from electronic health record lab measurements (LOINC Code: 704-7; OMOP Concept ID: 3013429). Unit of measurement is thousand/microliter. |
| Eosinophils (blood) | Blood counts | Continuous | Obtained from electronic health record lab measurements (LOINC Code: 711-2; OMOP Concept ID: 3028615). Unit of measurement is thousand/microliter. |
| RDW (blood) | Blood counts | Continuous | Obtained from electronic health record lab measurements. (LOINC Code: 788-0; OMOP Concept ID: 3019897). Unit of measurement is percent. |
| Hematocrit (blood) | Blood counts | Continuous | Obtained from electronic health record lab measurements (LOINC Code: 4544-3; OMOP Concept ID: 3023314). Unit of measurement is percent. |
| Hemoglobin (blood) | Blood counts | Continuous | Obtained from electronic health record measurement files (LOINC Code: 718-7; OMOP Concept ID: 3000963). Unit of measurement is  g/dL. |
| RBC (blood) | Blood counts | Continuous | Obtained from electronic health record lab measurements (LOINC Code: 742-7;  OMOP Concept ID: 3033575). Unit of measurement is millions/microliter. |
| Monocytes (blood) | Blood counts | Continuous | Obtained from electronic health record lab measurements (LOINC Code: 742-7;  OMOP Concept ID: 3033575). Unit of measurement is thousand/microliter. |
| Platelets (blood) | Blood counts | Continuous | Obtained from electronic health record lab measurements (LOINC Code: 777-3; OMOP Concept ID: 3024929). Unit of measurement is thousand/microliter. |
| Neutrophils (blood) | Blood counts | Continuous | Obtained from electronic health record lab measurements (LOINC Code: 751-8; OMOP Concept ID: 3013650). Unit of measurement is thousand/microliter. |
| Breast Cancer (Female only) | Cancer | Categorical (Binary) | Any occurrence of ICD9/10 code corresponding with PheCode174.1 (ICD-9 174x, V10.3; ICD-10, C50.01, C50.011, C50.21, C50.31, C50.41, C50.51, C50.61, C50.81, C50.91x, Z85.3) OR Self-reported personal medical history of breast cancer indicated on survey response AND female sex assigned-at-birth.  ICD-9/10 codes indicating DCIS were excluded.  ICD-9/10 codes specific to male breast cancer were excluded. |
| Melanoma Skin Cancer | Cancer | Categorical (Binary) | Any occurrence of ICD9/10 code corresponding with PheCode 172.1 (ICD-9 172x, V10.82; ICD-10 C43x, D03x, Z85.82x).  No personal medical history survey responses were included as these did not distinguish between melanoma and non-melanoma skin cancers. |
| Prostate Cancer (male only) | Cancer | Categorical (Binary) | Any occurrence of ICD9/10 Code corresponding with PheCode185 (ICD-9:185, 233.4, V10.46; ICD-10 C61, D07.5) OR Self-reported personal medical history of prostate cancer indicated on survey response AND assigned male sex-at-birth. |
| Skin Cancer (melanoma and non-melanoma skin cancers) | Cancer | Categorical (Binary) | Any occurrence of ICD9/10 code corresponding with PheCode 172 (ICD-9 172, 173, 209.3, 232, V10.82, V10.83; ICD-10 C43-C44, D03x, D04x, Z85.82x) ORSelf-reported personal medical history of skin cancer on survey response (non-specific regarding type of skin cancer) |
| Atrial Fibrillation | Cardiometabolic | Categorical (Binary) | Any occurrence of ICD9/10CM code corresponding with PheCodes 427.21 & 427.22 (ICD-9 427.3, ICD-10 I48) or self-reported personal medical history of atrial fibrillation. |
| Coronary Artery Disease | Cardiometabolic | Categorical (Binary) | At least one ICD or CPT code below: ICD9 410x, 411x, 412x; ICD10 I22x, I23x, I24.1, I25.2 CPT 92920-92979 (PCI), 33533-33536, 33517-33523, 33510-33516 (CABG) or self-reported personal history of MI or CAD. |
| HDL | Cardiometabolic | Categorical (Binary) | Obtained from electronic health record lab measurement files for those with lipid measurements available (OMOP Concept IDs for lipid values: 37040799, 37053082, 37059768, 40772572, 37077580, 37027885, 40782589, 37039062, 37060031, 37024395, 37074494, 40795800, 37026687).^2^  No adjustment was made for concurrent statin use.^3^ Units of measurement are mg/dL. |
| High Cholesterol | Cardiometabolic | Categorical (Binary) | Any occurrence of ICD9/10CM code corresponding with PheCodes 272.1x (ICD-9 272.0-272.2, 272.3, ICD-10 E78.0-E78.2, E78.4) or self-reported personal medical history of high cholesterol. |
| HTN | Cardiometabolic | Categorical (Binary) | Any occurrence of ICD9/10CM code corresponding with PheCodes 401x (ICD-9 401-405, 997.91, ICD-10 I10-I13, I15, I16, I97.3, N26.2) or self-reported personal medical history of high blood pressure. |
| LDL-C | Cardiometabolic | Categorical (Binary) | Obtained from electronic health record lab measurement files for those with lipid measurements available (OMOP Concept IDs for lipid values: 37040799, 37053082, 37059768, 40772572, 37077580, 37027885, 40782589, 37039062, 37060031, 37024395, 37074494, 40795800, 37026687).^2^  The LDL cholesterol was divided by 0.7 for persons with EHR record for concurrent statin use.^3^ Units of measurement are mg/dL. |
| Systolic and Diastolic Blo0d Pressure | Cardiometabolic | Categorical (Binary) | Average of three readings of systolic and diastolic blood pressure measured at the study visit. These measures were not adjusted for concurrent antihypertensive use. Units of measurement are mmHg. |
| Stroke (ischemic and non-ischemic stroke) | Cardiometabolic | Categorical (Binary) | Any occurrence of ICD9/10 Code corresponding with PheCodes 430x, 433.11, 433.21, or 433.6 (ICD-9 346.6, 430-432, 433.01, 433.x1, 434.x1, 436;ICD-10 I60-I63, G43.6) OR  Self-reported personal medical history medical history of stroke indicated on survey response. |
| Total Cholesterol | Cardiometabolic | Categorical (Binary) | Obtained from electronic health record lab measurement files for those with lipid measurements available. (OMOP Concept IDs for lipid values: 37040799, 37053082, 37059768, 40772572, 37077580, 37027885, 40782589, 37039062, 37060031, 37024395, 37074494, 40795800, 37026687).^2^ The Total Cholesterol was divided by 0.8 for persons with EHR records for concurrent statin use.^3 ^Units of measurement are mg/dL. |
| Triglycerides | Cardiometabolic | Categorical (Binary) | Obtained from electronic health record lab measurement files for those with lipid measurements available. (OMOP Concept IDs for lipid values: 37040799, 37053082, 37059768, 40772572, 37077580, 37027885, 40782589, 37039062, 37060031, 37024395, 37074494, 40795800, 37026687).^2 ^No adjustment was made for concurrent statin use.^3^ Units of measurement are mg/dL. |
| Type II DM^4^ | Cardiometabolic | Categorical (Binary) | Meets any of the following case definitions: 1 (or more) diagnosis codes for Type 2 diabetes (T2D) AND 1 (or more) drug exposure for Type 2 diabetes medications EXCLUDE 1 (or more) diagnosis codes for Type 1 diabetes (T1D), OR 1 (or more) diagnosis codes AND 2 (or more) drug exposures for Type 1 diabetes and Type 2 diabetes medications with Type 2 diabetes drug exposure occurring ≥1 day before Type 1 diabetes drug exposure EXCLUDE 1 (or more) diagnosis codes for Type 1 diabetes, OR 2 (or more) diagnosis codes for Type 2 diabetes AND 1 (or more) drug exposure for Type 1 diabetes medication EXCLUDE 1 (or more) diagnosis codes for Type 1 diabetes OR 1 (or more) drug exposures for Type 2 diabetes medications AND 1 (or more) lab results EXCLUDE 1 (or more) diagnosis codes for Type 1 diabetes. |
| Venous Thromboembolism | Cardiometabolic | Categorical (Binary) | Any occurrence of ICD9/10CM code corresponding with PheCodes 415.1 (pulmonary embolism) and 452.2 (deep vein thrombosis) (ICD-9 415.12-415.19, 453.4-453.5, 453.7-453.8, ICD-10 I26, I82.2, I82.4-I82.7, I82A/B/C) or self-reported personal medical history of DVT or PE. |
| Asthma | Other conditions | Categorical (Binary) | Any occurrence of ICD9/10CM code corresponding with Phecode 495 (ICD-9 493x or ICD-10CM J45x) OR self-reported personal medical history of asthma indicated on survey response. |
| Current Cigarette Smoker | Other conditions | Categorical (Binary) | Cases are respondents who indicated that they currently smoke cigarettes “Every Day” or “Some Days” on the Lifestyle Survey; controls include those who  denied smoking currently or in their lifetime.  Those who did not complete the lifestyle survey or indicated “Don’t Know,” “Prefer Not To Answer,” or skipped the question were excluded. |
| Depression | Other conditions | Categorical (Binary) | Any occurrence of ICD9/10CM code corresponding with Phecode 296.2x (ICD-9 296.2x-296.3x, 311; ICD-10CM: F32x, F33x) OR self-reported personal medical history of depression indicated on survey response. |
| Glaucoma | Other conditions | Categorical (Binary) | Any occurrence of ICD9/10CM code corresponding with PheCodes 365 (ICD-9 365, ICD-10 H40, H42) or self-reported personal medical history of glaucoma. |
| Gout | Other conditions | Categorical (Binary) | Any occurrence of ICD9/10CM code corresponding with PheCodes 274.1x (ICD-9 274, ICD-10 M10, M1A) or self-reported personal medical history of gout. |
| Hypothyroidism | Other conditions | Categorical (Binary) | Any occurrence of ICD9/10CM code corresponding with PheCode 244x (ICD-9 243, 244, ICD-10 E00-E03, E89.0) or self-reported personal medical history of hypothyroidism. |
| Migraines | Other conditions | Categorical (Binary) | Any occurrence of ICD9/10CM code corresponding with Phecode 340 (ICD-9 339.0x, 346x or ICD-10CM G43, G44.0x) or self-reported medical history of migraine. |
| Osteoporosis | Other conditions | Categorical (Binary) | Any occurrence of ICD9/10CM code corresponding with PheCodes 743.1x (ICD-9 733.0, ICD-10 M81) or self-reported personal medical history of osteoporosis. |
| Rheumatoid Arthritis (RA) | Other conditions | Categorical (Binary) | Any occurrence of ICD9/10CM code corresponding with PheCodes 714 (ICD-9 714, ICD-10 M05-M06) or self-reported personal medical history of rheumatoid arthritis. |

^1^Citation: Natarajan P, Pampana A, Graham SE, et al. Chromosome Xq23 is associated with lower atherogenic lipid concentrations and favorable cardiometabolic indices. Nat Commun. 2021 Apr 12;12(1):2182. doi: 10.1038/s41467-021-22339-1. PMID: 33846329; PMCID: PMC8042019.

^2^Consistent with the All of Us Research Program’s demonstration project entitled, “[Demo - Siloed Analysis of All of Us and UK Biobank Genomic Data](https://workbench.researchallofus.org/workspaces/aou-rw-6ef5a3b8/siloedanalysisofallofusandukbiobankgenomicdata/data),” available on the researcher workbench with code deposited at: https://github.com/all-of-us/ukb-cross-analysis-demo-project

^3^Lipids measurements were adjusted for concurrent statin use as is typical in prior GWAS (e.g. PMIDs: 30939045, 33967275)

^4^Based on All of Us Demonstration project, which replicated DM2 phenotype algorithm that was obtained from the eMERGE network. Citation: Jennifer Pacheco and Will Thompson. Northwestern University. Type 2 Diabetes Mellitus. PheKB; 2012 Available from: [https://phekb.org/phenotype/18](https://phekb.org/phenotype/18)

###### **Supplementary Table 14. Phenotype Definitions and Descriptions from the Genes and Health cohort.** 

For binary phenotypes, cases were curated as per the definitions below.  Controls were defined as those who did not meet the case definition, and who had consented to linking of EHR data.

Continuous measures included either body measurements, which were directly measured at the participant’s study visit, or laboratory measurements, obtained from electronic health record data.  For all laboratory measurements, if a participant had multiple measurements available, the average was used after cleaning.

| **Trait** | **Trait Subtype** | **Variable Type** | **Definition** |
| --- | --- | --- | --- |
| BMI | Anthropometric | Continuous | Calculated BMI from participant measurements obtained during the study visit.  Patients who indicated pregnancy at enrollment were excluded. Unit of measure is kg/m^2. |
| Height | Anthropometric | Continuous | From participant measurement data obtained during the study visit. Unit of measure is cm. |
| Basophils (blood) | Blood counts | Continuous | Obtained from electronic health record lab measurements. Unit of measurement is thousand/microliter. |
| Eosinophils (blood) | Blood counts | Continuous | Obtained from electronic health record lab measurements. Unit of measurement is thousand/microliter. |
| Monocytes (blood) | Blood counts | Continuous | Obtained from electronic health record lab measurements. Unit of measurement is thousand/microliter. |
| Neutrophils (blood) | Blood counts | Continuous | Obtained from electronic health record lab measurements. Unit of measurement is thousand/microliter. |
| Platelets (blood) | Blood counts | Continuous | Obtained from electronic health record lab measurements. Unit of measurement is thousand/microliter. |
| Red blood cell (blood) | Blood counts | Continuous | Obtained from electronic health record lab measurements. Unit of measurement is millions /microliter. |
| White blood cell (blood) | Blood counts | Continuous | Obtained from electronic health record lab measurements. Unit of measurement is thousand/microliter. |
| Hematocrit (blood) | Blood counts | Continuous | Obtained from electronic health record lab measurements. Unit of measurement is percent. |
| RDW (blood) | Blood counts | Continuous | Obtained from electronic health record lab measurements. Unit of measurement is percent. |
| Albumin (plasma/serum) | Biochemistry | Continuous | Obtained from electronic health record lab measurements. Unit of measurement is g/dL. |
| ALP (plasma/serum) | Biochemistry | Continuous | Obtained from electronic health record lab measurements. Unit of measurement is units per liter. |
| ALT/SGPT (plasma/serum) | Biochemistry | Continuous | Obtained from electronic health record lab measurements. Unit of measurement is units per liter. |
| AST/SGOT (plasma/serum) | Biochemistry | Continuous | Obtained from electronic health record lab measurements. Unit of measurement is units per liter. |
| Blood Urea Nitrogen (BUN) (plasma/serum) | Biochemistry | Continuous | Obtained from electronic health record lab measurements. Unit of measurement is mg/dL. |
| Creatinine (plasma/serum) | Biochemistry | Continuous | Obtained from electronic health record lab measurements. Unit of measurement is mg/dL. |
| A1c (blood) | Biochemistry | Continuous | Obtained from electronic health record lab measurements. No adjustment for concurrent diabetes medication use.  Unit of measure is %. |
| Total Protein  (plasma/serum) | Biochemistry | Continuous | Obtained from electronic health record lab measurements. Unit of measurement is g/dL. |
| Leukocytes (blood) | Blood counts | Continuous | Obtained from electronic health record lab measurements. Unit of measurement is thousand/microliter. |
| Hemoglobin (blood) | Blood counts | Continuous | Obtained from electronic health record lab measurements. Unit of measurement is g/dL. |
| RBC (blood) | Blood counts | Continuous | Obtained from electronic health record lab measurements (LOINC Code: 742-7;  OMOP Concept ID: 3033575). Unit of measurement is millions/microliter. |
| Breast Cancer (Female only) | Cancer | Categorical (Binary) | Any occurrence of ICD-10 code corresponding with C50.01, C50.1, C50.2, C50.3, C50.4, C50.5, C50.6, C50.8, C50.9,C50x,D05.0,D05.1,D05.7,D05.9,D05x,D48.6,Z85.3 OR SNOMED codes 2985005, 30566004, 35232005, 41919003, 54666007, 64524002, 78197004, 82591005, 82711006, 93680004, 93681000, 93924008, 93925009, 109888004, 109889007, 126926005, 188147009, 188151006, 188152004, 188153009, 188154003, 188155002, 188156001, 188157005, 188159008, 188163001, 188168005, 189336000, 254837009, 269497004, 314955001, 372064008, 372095001, 429087003, 703545003, 72223700 |
| Melanoma Skin Cancer | Cancer | Categorical (Binary) | Any occurrence of ICD10 code corresponding with ICD-10 C43x, D03x, Z85.82x OR SNOMED codes 915007, 1556006, 2092003, 2142002, 16034002, 16974005, 18450009, 21165006, 37138001, 39274007, 39896009, 40244008, 44474009, 50542000, 50813003, 51757004, 55320002, 61217001, 68827007, 70594002, 75931002, 83284001, 88082008, 93209006, 93217003, 93225001, 93643005, 93651008, 93655004 106243009, 109266006, 109267002, 109270003, 109272006, 109274007, 109276009, 93223008, 109278005 109284008, 109290007, 109294003, 115223002, 127330008, 161432005, 177281002, 188030005, 188032002, 188033007, 188034001, 188038003 188040008, 188042000, 188044004, 188045003, 188046002, 188049009, 188050009, 188051008, 188052001, 188054000, 188055004, 188060000, 188061001, 188062008, 188063003, 188064009, 188065005, 188067002, 188068007, 188069004, 188070003, 188071004, 188072006 188073001, 188074007, 188075008, 188076009, 188077000, 189324006, 231834005, 269579005, 269580008, 274087000, 302836005, 310498001, 313248004, 315036008, 315230006, 372130007, 956331000000107, 956351000000100, 956371000000109, 956391000000108, 956411000000108, 956431000000100, 956451000000107, 956471000000103, 956511000000107, 956531000000104, 95655100000010. |
| Prostate Cancer (male only) | Cancer | Categorical (Binary) | Any occurrence of ICD10 Code corresponding with ICD-10 C61x, D07.5, OR SNOMED codes 81232004, 176106009, 176261008, 176262001, 176263006, 369775001, 369777009, 385377005, 394932008, 399068003, 428262008 |
| Skin Cancer (melanoma and non-melanoma skin cancers) | Cancer | Categorical (Binary) | Any occurrence of ICD10 code corresponding with ICD-10 C43-C44, D03x, D04x, Z85.82x OR SNOMED codes 1338007, 6641007, 30649006, 32272007, 37304002, 43369006, 54734006, 56665009, 57141000, 61098004, 64000002, 93956001, 94000008 94008001, 94011000, 94012007, 94013002, 94016005, 94020009, 94023006, 94024000, 94026003, 94027007, 94028002, 94029005, 94030000, 94032008, 94034009, 94036006, 94039004, 94041003, 94042005, 94044006, 94047004, 94081005, 109264009, 126488004, 127570002 128636006, 134152008, 188089003, 188090007, 188091006, 188095002, 188099008, 188100000, 188102008, 188103003, 188107002, 188110009 188119005, 188121000, 188122007, 188125009, 188127001, 188132000, 188133005, 188135003, 188138001, 231829006, 254651007, 254701007, 266987004, 275265005, 276799004, 277156006, 301889008, 310599006, 311779007, 313249007, 313250007, 372126009, 372130007 400155002, 402529002, 403909004, 428796002, 44313600 |
| Atrial Fibrillation | Cardiometabolic | Categorical (Binary) | Any occurrence of ICD10 code corresponding with ICD-10 I48x or SNOMED codes 5370000, 49436004, 195080001, 282825002, 427665004, 44005900 |
| Coronary Artery Disease | Cardiometabolic | Categorical (Binary) | At least one ICD10 I22x, I23x, I24.1, I25 OR operation codes K401, K402, K403, K404, K411, K451, K452, K453, K454, K455, K491, K492, K499, K502, K751, K752, K753, K754, K758, K759 OR SNOMED codes 1755008, 22298006, 54329005, 57054005, 65547006, 70211005, 70422006, 73795002, 233838001, 304914007, 401303003, 401314000 |
| HDL | Cardiometabolic | Categorical (Binary) | Obtained from electronic health record lab measurement files for those with lipid measurements available (OMOP Concept IDs for lipid values: 37040799, 37053082, 37059768, 40772572, 37077580, 37027885, 40782589, 37039062, 37060031, 37024395, 37074494, 40795800, 37026687). No adjustment was made for concurrent statin use. Units of measurement are mg/dL. |
| HTN | Cardiometabolic | Categorical (Binary) | Any occurrence of ICD-10 I10-I13, I15, I16, I97.3, N26.2 OR operation code X82 OR SNOMED codes 193003, 264008, 1201005, 6962006, 24184005, 31992008, 36221001, 36315003, , 38341003, 38481006, 48146000, 49220004, 50490005, 54225002, 56218007, 59621000, 64715009, 65443008, 66052004, 66610008, 73410007, 77970009, 78975002, 83105008, 86041002, 86234004, 89242004, 123799005, 161501007, 162659609, 169465000, 170574005, 170577003, 170578008, 170586008, 170587004, 185724004, 190507007, 194779001, 194780003, 194781904, 194783001, 194785008, 194788005, 194791005, 198997005, 275516004, 293495006, 302192008, 308116003, 401047000, 401048005, 401117004, 401118009, 407567007, 220901000000101, 299561000000105 |
| LDL-C | Cardiometabolic | Categorical (Binary) | Obtained from electronic health record lab measurement files for those with lipid measurements available. Units of measurement are mg/dL. |
| Systolic and Diastolic Blo0d Pressure | Cardiometabolic | Categorical (Binary) | Average of three readings of systolic and diastolic blood pressure measured at the study visit. These measures were not adjusted for concurrent antihypertensive use. Units of measurement are mmHg. |
| Stroke (ischemic and non-ischemic stroke) | Cardiometabolic | Categorical (Binary) | Any occurrence of ICD-10 I60-I63, G43.6 OR SNOMED codes 20059004, 71444005, 195216008, 195217094, 195233001, 30691006, 307766002, 307767006, 413102000, 43250400 |
| Total Cholesterol | Cardiometabolic | Categorical (Binary) | Obtained from electronic health record lab measurement files for those with lipid measurements available. Units of measurement are mg/dL. |
| Triglycerides | Cardiometabolic | Categorical (Binary) | Obtained from electronic health record lab measurement files for those with lipid measurements available. No adjustment was made for concurrent statin use. Units of measurement are mg/dL. |
| Type II DM | Cardiometabolic | Categorical (Binary) | Any occurrence of ICD10 E11x OR SNOMED codes 44054006, 237599002, 313436004, 422099009, 703138006 |
| Venous Thromboembolism | Cardiometabolic | Categorical (Binary) | Any occurrence of ICD-10 I26, I82.2, I82.4-I82.7, I82A/B/C or SNOMED codes 14246007, 17920008, 21258007, 40198004, 42861008, 59282003, 82385007, 89980009, 95455008, 128053003, 134399007, 161508001, 161512007, 192753009, 192754003, 192755002, 192757005, 192759008, 192760003, 192761004, 192769002, 192770001, 192771002, 195229008, 195230003, 195394007, 195410000, 195411001, 195412008, 195414009, 195425000, 195426004, 195427008, 195437003, 195438008, 197001004, 213220000, 266267005, 276494008, 281595001, 297156001, 404223003, 413956008, 438647008, 438773007, 443210003, 710167004, 770111009, 492161000000109, 503421000000105, 503451000000100, 503481000000106, 864191000000104, 864211000000100, 86961100000010 |
| Asthma | Other conditions | Categorical (Binary) | Any occurrence of ICD-10 code corresponding with ICD-10CM J45x OR SNOMED codes 63088003, 195949008, 195967001, 195977004, 233678006, 233679003, 424643009, 708038006, 708090002, 734904007 233683003, 266361008, 370218001, 370221004, 389145006, 405944004 |
| Current Cigarette Smoker | Other conditions | Categorical (Binary) | Cases are respondents who indicated that they currently smoke cigarettes “Every Day” or “Some Days” on the Lifestyle Survey. Those who did not complete the lifestyle survey or indicated “Don’t Know,” “Prefer Not To Answer,” or skipped the question were excluded. |
| Depression | Other conditions | Categorical (Binary) | Any occurrence of ICD-10 F32x, F33x OR SNOMED codes 832007, 15639000, 19527009, 28475009, 35489007, 36474008, 36923009, 40568001, 46206005, 46244001, 58703003, 70747007, 73867007, 75084000, 76105009, 78667006, 79298009, 83458005, 84760002, 87414006, 87512008, 161469008, 162722001, 191455000, 191459006, 191466007, 191604000, 191610000, 191611001, 191613003, 191616006, 191659001, 191676002, 192046006, 192049004, 192079006, 192080009, 192362008, 231485007, 231499006, 231500002, 231504006, 231542000, 247803002, 268621008, 272022009, 274948002, 300706003, 310495003, 310496002, 310497006, 366979004, 386782008, 394924000, 395072006, 401174001, 413169006, 413170007, 413972000, 413973005, 413974004, 415044007, 698957003, 251000119105, 166291000000108, 166481000000107, 196381000000100, 199111000000100, 361761000000106, 713831000000108, 716421000000103, 716681000000100, 716961000000102, 717211000000107, 717261000000109, 755321000000106, 755331000000108, 764611000000100, 764691000000109, 790961000000101 |
| Glaucoma | Other conditions | Categorical (Binary) | Any occurrence of ICD-10 H40, H42 or operation codes C50x, C60x, C61x OR SNOMED codes 1207009, 1654001, 19144002, 23986001, 29369005, 29538005, 30041005, 32893002, 33647009, 34623005, 37155002, 46168003, 50485007, 65460003, 66990007, 67733005, 68241007, 71111008, 77075001, 84333006, 84494001, 95717004, 111514006, 170946006, 193533000, 193534006, 193546005, 193549003, 193552006, 193553001, 193555008, 193556009, 193561006, 193562004, 172586003, 204113001, 206248004, 232080006, 232086000, 267625001, 275477002, 302895007, 392288006, 415273001, 426877004, 428494000, 234611000000100, 236891000000101, 792741000000100 |
| Gout | Other conditions | Categorical (Binary) | Any occurrence of ICD-10 M10, M1A OR SNOMED codes 24595009, 48440001, 90560007, 190842000, 201666003, 201669005, 428839004, 924311000000000 |
| Migraines | Other conditions | Categorical (Binary) | Any occurrence ICD-10 G43, G44.0x OR SNOMED codes 4473006, 23186000, 37796009, 49605003, 56097005, 59292006, 75879005, 83351003, 95655001, 161481007, 193030005, 193039006, 230462002, 230467008, 408381007, 608837004, 78138100000010 |
| Osteoporosis | Other conditions | Categorical (Binary) | Any occurrence of ICD-10 M81 OR SNOMED codes 14651005, 18040001, 53174001, 64859006, 102447009, 203429007, 203433000, 203434006, 203437004, 203438009, 203444008, 203445009, 203446005, 203447001, 203448006, 203450003, 203451004, 203452006, 203453001, 203657009, 240198062, 309745002, 311806008, 311890007, 311891006, 390833005, 391003008, 391004002, 391006000, 391007009, 391008004, 391009007, 391010002, 391011003, 391012005, 391013000, 391017004, 391020007, 391022004, 391023009, 391030003, 391060008, 391065003, 391070005, 391075000, 391081008, 391092009, 440100002, 443165006, 473120007, 306171000000106, 505141000000108, 505171000000102, 711341000000106, 711391000000101, 96587100000010 |
| Rheumatoid Arthritis (RA) | Other conditions | Categorical (Binary) | Any occurrence of ICD-10 M05-M06 OR SNOMED codes 9631008, 10713006, 23060008, 28880005, 33719002, 57160007, 69896004, 84017003, 161567008, 164454008, 164456005, 193180002, 193250002, 195136004, 201764007, 201766009, 201767000, 201768005, 201769002, 201770001, 201771002, 201772009, 201773004, 201774005, 201775006, 201775004, 201776007, 201777003, 201778008, 201779000, 239791005, 239792003, 2399308, 239920006, 201780002, 201781003, 201783000, 201784006, 201785007, 201791009, 287006005, 308143008, 398640008, 398726004, 400054000, 429192004, 441870009, 763259004, 231041000000107, 847261000000104, 882321000000105, 882401000000106, 882421000000102, 882441000000109, 882461000000105, 882481000000101 |
